# Crystal structure of 4′-allyl-4,5,6,7,2′,7′-hexa­chloro­fluorescein allyl ester unknown solvate

**DOI:** 10.1107/S2056989017018163

**Published:** 2018-01-01

**Authors:** Lili Wang, Alexander S. Filatov, Gregory S. Engel

**Affiliations:** a Department of Chemistry, James Franck Institute, Institute for Biophysical Dynamics, University of Chicago, 5735 South Ellis ave, Chicago, IL 60637, USA

**Keywords:** crystal structure, fluorescein, hydrogen bonding, Cl⋯π inter­action

## Abstract

In the title compound, 4′-allyl-4,5,6,7,2′,7′-hexa­chloro­fluorescein allyl ester {systematic name: prop-2-en-1-yl 2,3,4,5-tetra­chloro-6-[2,7-di­chloro-6-hy­droxy-3-oxo-4-(prop-2-en-1-yl)-3*H*-xanthen-9-yl]benzoate}, C_26_H_14_Cl_6_O_5_, accompanied by unknown solvate molecules, the dihedral angle between the xanthene ring system (r.m.s. deviation = 0.046 Å) and the penta­substituted benzene ring is 71.67 (9)°. Both allyl groups are disordered over two sets of sites in statistical ratios. The scattering contributions of the disordered solvent mol­ecules (both Ph_2_O and CHCl_3_, as identified by NMR) were removed with the *PLATON* SQUEEZE algorithm [Spek (2015 ▸). *Acta Cryst.* C**71**, 9–18]. In the crystal, tetra­meric supra­molecular aggregates linked by O—H⋯O hydrogen bonds occur; these further inter­act with neighboring aggregates through C—Cl⋯π inter­actions arising from the benzene rings, forming infinite two-dimensional sheets. Each C_6_Cl_4_ ring shifts in the direction perpendicular to the two-dimensional sheet, exhibiting a helical chain in which every C_6_Cl_4_ ring is utilized as both a donor and an acceptor of Cl⋯π contacts. Thus, these two-dimensional sheets pack in a helical fashion, constructing a three-dimensional network.

## Abstract 2

In the crystal, tetra­meric supra­molecular aggregates linked by O—H⋯O hydrogen bonds occur; these further inter­act with neighboring aggregates through C—Cl⋯π inter­actions arising from the benzene rings, forming infinite two-dimensional sheets. Each C_6_Cl_4_ ring shifts in the direction perpendicular to the two-dimensional sheet, exhibiting a helical chain in which every C_6_Cl_4_ ring is utilized as both a donor and an acceptor of Cl⋯π contacts. Thus, these two-dimensional sheets pack in a helical fashion, constructing a three-dimensional network.

## Chemical context   

Fluorescein derivatives have been widely used in chemical and biological research. The high fluorescence quantum yields and excellent photostability of these dyes make them attractive as fluorescent labels for macromolecules such as proteins (Giepmans *et al.*, 2006 ▸) or DNA (Li *et al.*, 1995 ▸). Fluorescein derivatives also exhibit tunable optical transitions in the visible range and high molar extinction coefficients, making them suitable for optical laser and dye-sensitized solar cell applications (Pepe *et al.*, 2016 ▸). Understanding the properties of these fluorescein derivatives, especially their bonding abilities at certain local environments, is essential for designing and utilizing these compounds. Detailed crystal structure determinations of fluorescein derivatives can reveal their bonding/packing properties, providing valuable insights in directing future mol­ecular engineering design and chemical and biological applications. Until recently, the different forms of fluorescein could only be obtained as microcrystalline powders and the first crystal structure determination of free fluorescein came from powder diffraction data analysis (Tremayne *et al.*, 1997 ▸). It was then followed by a number of single crystal X-ray structural analyses of fluorescein derivatives. For several recent examples, see Christianson & Gabbaï (2016 ▸), Sezukuri *et al.* (2016 ▸), and Dufresne *et al.* (2007 ▸).

The title compound, 4′-allyl-4,5,6,7,2′,7′-hexa­chloro­fluorescein allyl ester, is an important inter­mediate in the synthetic route of structurally flexible fluorescein heterodimers that were recently published by us (Wang *et al.*, 2017 ▸). Such heterodimers were designed to test the engineering principle of quantum coherences in artificial light-harvesting systems. Herein, we present the crystal structure of the title compound, which reveals the importance of Cl⋯π inter­actions in the solid state.
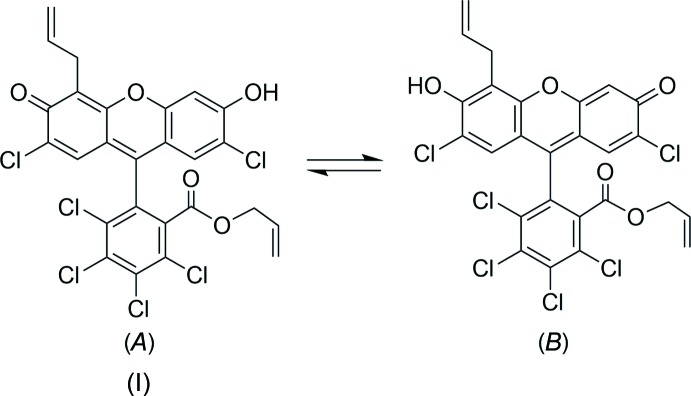


## Structural commentary   

**Figure 1:**
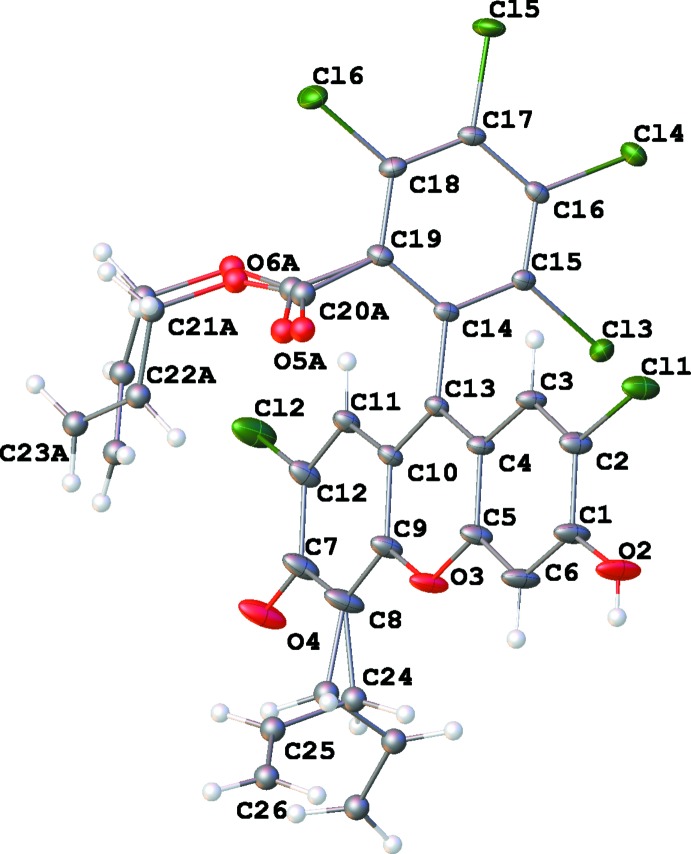
The mol­ecular structure of the title compound with 40% displacement ellipsoids. H atoms as well as atoms of the disordered allyl groups are shown as spheres of arbitrary radius.

**Figure 2:**
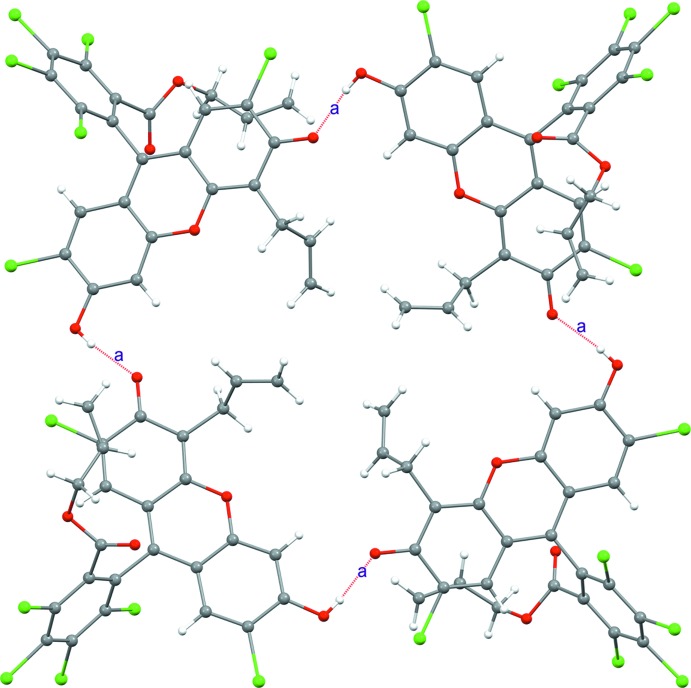
A tetra­meric hydrogen-bonded aggregate formed by the title compound: O2—H2⋯O4 bonds are labeled as ‘a’. The assemblage has 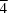 symmetry.

The mol­ecular structure of the title compound is shown in Fig. 1 ▸. The structure consists of a xanthene ring system, a perchlorinated phenyl ring and two allyl groups; one is located at the periphery of the xanthene ring while the other is linked to the six-membered ring through the carboxyl­ate linker (atom O6). The phenyl plane inclines from the xanthene plane by about 73 ° [the C4—C13—C14—C15 torsion angle is 72.7 (3)°]. The unusual unsymmetrical substitution pattern on the xanthene ring of the title compound leads to the possibility of having different tautomers as depicted in the Scheme. Unsymmetrically substituted fluoresceins have previously been reported, but until now all related structural reports showed only their spiroxanthene isomeric forms (Hou *et al.*, 2012 ▸; Swamy *et al.*, 2006 ▸; Wang *et al.*, 2005 ▸), thwarting a direct comparison with this study. While the title compound may exist as a mixture of exchanging tautomers (*A*) and (*B*) in solution, the solid-state structure is better described as tautomer (*A*) based on the bond-length distribution. For example, the bond lengths for C7—O4 [1.251 (3) Å] and C1—O2 [1.326 (3) Å] are consistent with a C=O double bond and a C—O single bond, respectively. The bond lengths of C8—C9, C10—C13, and C11—C12, which are 1.359 (4), 1.373 (3), and 1.347 (3) Å, respectively, are significantly shorter than C7—C12 and C10—C11 [1.459 (4) and 1.429 (3) Å, respectively], suggesting that the former are of a double-bond character. It should be noted here that this tautomer may not represent the thermodynamically more stable tautomer that may exist in the gas phase, because this form may be stabilized by the formation of tetra­meric aggregates through inter­molecular O2—H2⋯O4 bonds as discussed below (Table 1 ▸, Fig. 2 ▸).

## Supra­molecular features   

**Figure 3:**
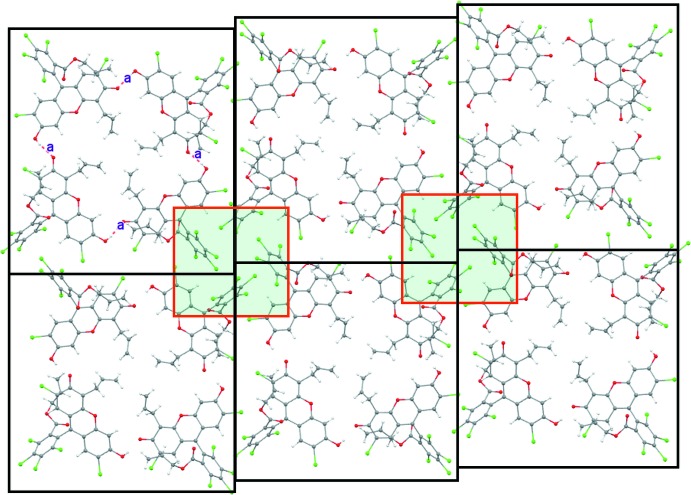
Infinite sheets formed by neighboring tetra­meric aggregates *via* Cl⋯π inter­actions. The aggregates are shown as large black squares and the inter­molecular inter­actions between them are shown as small red squares with a semi-transparent green background.

**Figure 4:**
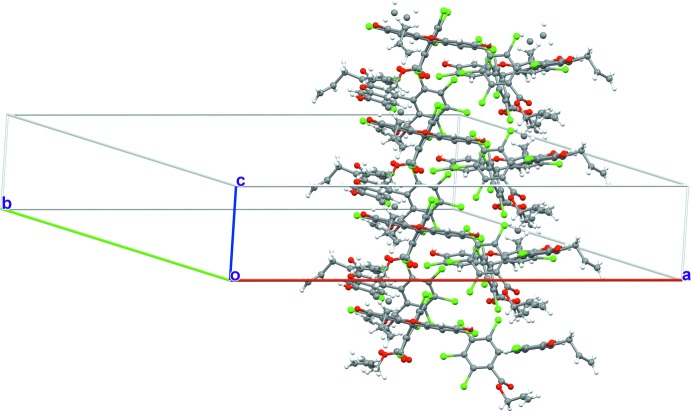
Three-dimensional packing diagram of the title compound.

In the crystal, the title compound forms tetra­meric aggregates linked by O2—H2⋯O4 hydrogen bonds, as shown in Fig. 2 ▸. The allyl groups sit inside the pocket formed by the hydrogen bonds and are not engaged in any particular inter­molecular inter­actions (only one disorder component is shown). The tetra­meric aggregates further inter­act with neighboring aggregates through Cl⋯π inter­actions of dangling C_6_Cl_4_ rings forming infinite two-dimensional sheets, as shown in Fig. 3 ▸. Each of the C_6_Cl_4_ rings accepts two edge-on Cl⋯C short contacts from an adjacent C_6_Cl_4_ unit [Cl4⋯C16 = 3.398 (3); Cl5⋯C18 = 3.333 (3) Å]. When viewed along the two-dimensional sheet located in the *ab* plane, it may be noted that each –C_6_Cl_4_ ring is in fact shifted in the direction perpendic­ular to the two-dimensional sheet. These C_6_Cl_4_ rings thus exhibit a helical chain in which every C_6_Cl_4_ ring is utilized as both a donor and an acceptor of Cl⋯π contacts. Thus, several layers of the tetra­meric aggregates are further packed in a helical manner in the third dimension along the *c* axis, constructing a three-dimensional network, as shown in Fig. 4 ▸.

## Database survey   

A search of the Cambridge Structural Database (CSD, Version 5.38, update May 2017; Groom *et al.*, 2016 ▸) indicated that several fluorescein derivatives with halogen substituents on the xanthene ring have been reported (Cody, 1987 ▸; Willner *et al.*, 1992 ▸; Harrison *et al.*, 2007 ▸; Quint *et al.*, 2016 ▸). However, there was only one structural report on fluorescein derivatives that contains a tetra­chloro-substituted phenyl unit (CCDC refcode KUFTUA; Willner *et al.*, 1992 ▸), and there were no structural reports on hexa­chlorinated fluorescein derivatives. While the hydroxyl groups on the xanthene rings of fluorescein derivatives have been reported to engage in hydrogen bonds (Abrahams *et al.*, 2009 ▸), to the best of our knowledge, the tetra­meric aggregation motif in this report has not been found previously for fluorescein derivatives.

## Synthesis and crystallization   

4,5,6,7,2′,7′-Hexa­chloro­fluorescein diallyl ether ester was synthesized following a literature method (Wang *et al.*, 2017 ▸). 4,5,6,7,2′,7′-Hexa­chloro­fluorescein diallyl ether ester (500 mg) in diphenyl ether (5 ml) was heated in a sealed tube at 443 K under N_2_ overnight. The homogeneous mixture was then cooled to room temperature, transferred to a scintillation vial, and diluted with CHCl_3_ (5 ml). Red prismatic crystals of the title compound formed slowly from this mixture at room temperature within three months, yield: 52%. This crystalline material contained 0.3 equiv. of diphenyl ether and *ca* 0.1 equiv of CHCl_3_, as determined by ^1^H NMR integration. Note that the qu­antity of CHCl_3_ could be underestimated because of the overly long *T*_1_ relaxation time of the H-CCl_3_ proton. The volatile nature of CHCl_3_ and the loss in the sample-dissolving process could also contribute to underestimation.

Data for the title compound: ^1^H NMR (500 MHz, CD_3_OD): δ 7.25 (*s*, 1H), 7.20 (*s*, 1H), 7.03 (*br s*, 1H), 5.96 (*ddt*, *J* = 16.9, 10.2, 6.5 Hz, 1H), 5.32 (*ddt*, *J* = 17.0, 10.4, 6.5 Hz, 1H), 5.17 (*dq*, *J* = 17.1, 1.7 Hz, 1H), 5.06–4.95 (*m*, 3H), 4.45–4.41 (*m*, 2H), 3.58 (*dt*, *J* = 6.4, 1.3 Hz, 2H).

Data for diphenyl ether: ^1^H NMR (500 MHz, CD_3_OD): δ 7.36–7.32 (*m*, 4H), 7.10 (*tt*, *J* = 7.5, 1.1 Hz, 2H), 6.98–6.96 (*m*, 4H).

Data for CHCl_3_: ^1^H NMR (500 MHz, CD_3_OD): δ 7.90 (*s*, 1H).

HRMS (ESI-TOF, positive ion, *m*/*z*): Calc. 618.9022 ([M + H]^+^), found 618.9015.

## Refinement   

Crystal data, data collection and structural refinement details are summarized in Table 2 ▸. Carbon-bound H atoms were placed in calculated positions (C—H = 0.95–0.98 Å) and were included in the refinement in the riding-model approximation, with *U*_iso_(H) set to 1.2–1.5*U*_eq_(C). The H atom of the hydroxyl group was found in a difference-Fourier map and freely refined [O—H = 0.74 (4) Å]. Most atoms except those of the allyl groups were refined anisotropically. Both allyl groups were found to be disordered and each disorder was individually modeled with the application of appropriate geometric (SADI) restraints or thermal parameters (EADP) constraints. The disorder was modelled over two positions (refined occupancies of 0.5:0.5 and 0.55:0.45). Similar distance soft restraints were used for the allyl groups. Hydrogen atoms were included in idealized positions for structure-factor calculations.

The crystal contained many disordered solvent mol­ecules located in several solvent-accessible voids. ^1^H NMR analysis of the crystalline material in MeOD revealed that both Ph_2_O and CHCl_3_ are present. The amount of Ph_2_O is qu­anti­fied to be 0.3 equiv. using the integrals for multiplets at δ 7.37–7.32 (4H), 7.12–7.07 (2H), and 6.98–6.96 (4H). The amount of CHCl_3_ is found to be approximately 0.1 equiv. using the integral for the singlet at δ 7.90. The amount of the CHCl_3_ is most probably underestimated owing to a very long T1 relaxation time of the HCCl_3_ proton and its loss in the sample during the dissolving process and crystals transfer. These results guided the disorder modeling of the allyl group pointing into the void as 0.5:0.5. The allyl group inside the void is poorly defined and could not be freely refined. Attempts to model the disordered solvent area were not successful, and the diffuse contribution to scattering was treated by application of the SQUEEZE routine (Spek, 2015 ▸) as implemented in *PLATON* (Spek, 2009 ▸) using the fab file construct: the SQUEEZE algorithm located four voids, centered at (0, 0.250, 0.625), (0, 0.750, 0.375), (0, 0.250, 0.875) and (0, 0.750, 0.125) with a volume of 860 Å^3^ and an electron count of 186 or approximately 47 electrons per mol­ecule of fluorescein. From the ^1^H NMR data, 0.3 equiv. of Ph_2_O and 0.2 equiv. of CHCl_3_ account for 39 electrons.

## Supplementary Material

Crystal structure: contains datablock(s) I. DOI: 10.1107/S2056989017018163/hb7719sup1.cif

Structure factors: contains datablock(s) I. DOI: 10.1107/S2056989017018163/hb7719Isup2.hkl

Supporting information file. DOI: 10.1107/S2056989017018163/hb7719Isup3.cdx

Supporting information file. DOI: 10.1107/S2056989017018163/hb7719Isup4.cml

CCDC reference: 1812489

Additional supporting information:  crystallographic information; 3D view; checkCIF report

## Figures and Tables

**Table 1:** Hydrogen-bond geometry (Å, °)

*D*—H⋯*A*	*D*—H	H⋯*A*	*D*⋯*A*	*D*—H⋯*A*
O2—H2⋯O4^i^	0.74 (4)	1.86 (4)	2.595 (3)	172 (4)

Symmetry code: (i) 
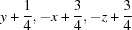
.

**Table 2:** Experimental details

Crystal data	
Chemical formula	C_26_H_14_Cl_6_O_5_
*M*_r_	619.07
Crystal system, space group	Tetragonal, *I*4_1_/*a*
Temperature (K)	100
*a*, *c* (Å)	41.432 (2), 7.7844 (6)
*V* (Å^3^)	13363.0 (18)
*Z*	16
Radiation type	Mo *K*α
μ (mm^−1^)	0.54
Crystal size (mm)	0.42 × 0.32 × 0.18

Data collection	
Diffractometer	Bruker D8 Venture
Absorption correction	Multi-scan (*SADABS*; Bruker, 2015 ▸)
*T*_min_, *T*_max_	0.803, 0.940
No. of measured, independent and observed [*I* > 2σ(*I*)] reflections	105222, 7959, 6656
*R*_int_	0.043
(sin θ/λ)_max_ (Å^−1^)	0.659

Refinement	
*R*[*F* ^2^ > 2σ(*F* ^2^)], *wR*(*F* ^2^), *S*	0.055, 0.156, 1.05
No. of reflections	7959
No. of parameters	327
No. of restraints	13
H-atom treatment	H atoms treated by a mixture of independent and constrained refinement
Δρ_max_, Δρ_min_ (e Å^−3^)	1.08, −0.69

Computer programs: *APEX3* and *SAINT* (Bruker, 2015 ▸), *SHELXT* (Sheldrick, 2015*a* ▸), *SHELXL2017* (Sheldrick, 2015*b* ▸) and *OLEX2* (Dolomanov *et al.*, 2009 ▸).

## supplementary crystallographic information

### Crystal data

**Table:**

C_26_H_14_Cl_6_O_5_	*D*_x_ = 1.231 Mg m^−^^3^
*M**_r_* = 619.07	Mo *K*α radiation, λ = 0.71073 Å
Tetragonal, *I*4_1_/*a*	Cell parameters from 9585 reflections
*a* = 41.432 (2) Å	θ = 2.2–27.9°
*c* = 7.7844 (6) Å	µ = 0.54 mm^−^^1^
*V* = 13363.0 (18) Å^3^	*T* = 100 K
*Z* = 16	Prism, red
*F*(000) = 4992	0.42 × 0.32 × 0.18 mm

### Data collection

**Table:**

Bruker D8 Venture diffractometer	6656 reflections with *I* > 2σ(*I*)
Detector resolution: 10.4167 pixels mm^-1^	*R*_int_ = 0.043
ω and phi scans	θ_max_ = 27.9°, θ_min_ = 2.2°
Absorption correction: multi-scan (SADABS; Bruker, 2015)	*h* = −54→54
*T*_min_ = 0.803, *T*_max_ = 0.940	*k* = −54→54
105222 measured reflections	*l* = −9→10
7959 independent reflections	

### Refinement

**Table:**

Refinement on *F*^2^	Primary atom site location: dual
Least-squares matrix: full	Secondary atom site location: difference Fourier map
*R*[*F*^2^ > 2σ(*F*^2^)] = 0.055	Hydrogen site location: mixed
*wR*(*F*^2^) = 0.156	H atoms treated by a mixture of independent and constrained refinement
*S* = 1.05	*w* = 1/[σ^2^(*F*_o_^2^) + (0.0825*P*)^2^ + 46.1798*P*] where *P* = (*F*_o_^2^ + 2*F*_c_^2^)/3
7959 reflections	(Δ/σ)_max_ = 0.001
327 parameters	Δρ_max_ = 1.08 e Å^−^^3^
13 restraints	Δρ_min_ = −0.69 e Å^−^^3^

### Special details

**Table:**

Geometry. All esds (except the esd in the dihedral angle between two l.s. planes) are estimated using the full covariance matrix. The cell esds are taken into account individually in the estimation of esds in distances, angles and torsion angles; correlations between esds in cell parameters are only used when they are defined by crystal symmetry. An approximate (isotropic) treatment of cell esds is used for estimating esds involving l.s. planes.

### Fractional atomic coordinates and isotropic or equivalent isotropic displacement parameters (Å^2^)

**Table:**

	*x*	*y*	*z*	*U*_iso_*/*U*_eq_	Occ. (<1)
Cl1	0.39543 (2)	0.48283 (2)	0.58350 (11)	0.03112 (17)	
Cl2	0.27839 (2)	0.26500 (2)	0.42276 (14)	0.0454 (2)	
Cl3	0.31144 (2)	0.41091 (2)	0.23233 (8)	0.02342 (15)	
Cl4	0.24970 (2)	0.45200 (2)	0.29555 (8)	0.02253 (15)	
Cl5	0.21385 (2)	0.44703 (2)	0.64520 (9)	0.02840 (16)	
Cl6	0.23918 (2)	0.40045 (2)	0.92801 (9)	0.03081 (17)	
O2	0.45019 (5)	0.44372 (5)	0.4967 (4)	0.0402 (6)	
H2	0.4637 (10)	0.4339 (10)	0.463 (5)	0.045 (11)*	
O3	0.39550 (4)	0.34387 (4)	0.4569 (3)	0.0305 (5)	
O4	0.34551 (5)	0.24470 (5)	0.3600 (4)	0.0467 (7)	
C1	0.42382 (6)	0.42571 (6)	0.5078 (4)	0.0286 (6)	
C2	0.39447 (6)	0.44152 (6)	0.5503 (4)	0.0247 (5)	
C3	0.36604 (6)	0.42505 (6)	0.5618 (3)	0.0212 (5)	
H3	0.346796	0.436173	0.591771	0.025*	
C4	0.36511 (6)	0.39153 (6)	0.5293 (3)	0.0189 (5)	
C5	0.39438 (6)	0.37617 (6)	0.4901 (4)	0.0242 (5)	
C6	0.42330 (6)	0.39261 (7)	0.4794 (4)	0.0313 (6)	
H6	0.442660	0.381395	0.452782	0.038*	
C7	0.34334 (7)	0.27382 (6)	0.4012 (5)	0.0320 (7)	
C8	0.37143 (7)	0.29388 (6)	0.4114 (5)	0.0348 (7)	
C9	0.36809 (6)	0.32553 (6)	0.4539 (4)	0.0244 (5)	
C10	0.33741 (5)	0.34042 (5)	0.4904 (3)	0.0179 (5)	
C11	0.30949 (6)	0.32027 (6)	0.4812 (3)	0.0211 (5)	
H11	0.288808	0.329055	0.505540	0.025*	
C12	0.31228 (6)	0.28889 (6)	0.4383 (4)	0.0262 (6)	
C13	0.33624 (5)	0.37283 (6)	0.5269 (3)	0.0169 (4)	
C14	0.30471 (5)	0.38966 (5)	0.5564 (3)	0.0158 (4)	
C15	0.29226 (5)	0.40932 (5)	0.4272 (3)	0.0162 (4)	
C16	0.26413 (5)	0.42701 (5)	0.4541 (3)	0.0167 (4)	
C17	0.24778 (5)	0.42433 (6)	0.6088 (3)	0.0187 (5)	
C18	0.25919 (6)	0.40341 (6)	0.7354 (3)	0.0198 (5)	
C19	0.28768 (6)	0.38606 (6)	0.7089 (3)	0.0192 (5)	
O5A	0.32942 (12)	0.37441 (15)	0.9111 (6)	0.0277 (12)*	0.515 (11)
O6A	0.28648 (16)	0.34190 (13)	0.8881 (7)	0.0337 (14)*	0.515 (11)
C20A	0.30476 (16)	0.36766 (16)	0.8496 (8)	0.0165 (16)*	0.515 (11)
C21A	0.2973 (2)	0.32063 (19)	1.0245 (10)	0.0398 (17)*	0.515 (11)
H21A	0.309106	0.333258	1.112086	0.048*	0.515 (11)
H21B	0.278333	0.310548	1.080631	0.048*	0.515 (11)
C22A	0.3178 (2)	0.29612 (19)	0.9564 (11)	0.058 (2)*	0.515 (11)
H22A	0.337240	0.303079	0.903352	0.069*	0.515 (11)
C23A	0.3123 (3)	0.2639 (2)	0.9600 (14)	0.077 (3)*	0.515 (11)
H23A	0.293156	0.255742	1.011623	0.092*	0.515 (11)
H23B	0.327502	0.249450	0.910923	0.092*	0.515 (11)
O5B	0.32477 (13)	0.36512 (17)	0.9121 (7)	0.0307 (13)*	0.485 (11)
O6B	0.27778 (16)	0.33861 (12)	0.8719 (7)	0.0290 (13)*	0.485 (11)
C20B	0.29915 (17)	0.36200 (18)	0.8455 (8)	0.0162 (17)*	0.485 (11)
C21B	0.2864 (2)	0.3138 (2)	1.0020 (11)	0.046 (2)*	0.485 (11)
H21C	0.302368	0.323054	1.082861	0.055*	0.485 (11)
H21D	0.266885	0.308056	1.068539	0.055*	0.485 (11)
C22B	0.2994 (4)	0.2857 (4)	0.928 (2)	0.111 (5)*	0.485 (11)
H22B	0.285565	0.271263	0.867772	0.134*	0.485 (11)
C23B	0.3325 (4)	0.2785 (5)	0.942 (3)	0.142 (7)*	0.485 (11)
H23C	0.346502	0.292773	1.001980	0.170*	0.485 (11)
H23D	0.340941	0.259395	0.891333	0.170*	0.485 (11)
C24	0.40398 (15)	0.28020 (16)	0.3449 (9)	0.0320 (9)*	0.5
H24A	0.400391	0.264945	0.248897	0.038*	0.5
H24B	0.418248	0.297825	0.304871	0.038*	0.5
C25	0.4188 (4)	0.2628 (3)	0.4996 (15)	0.104 (3)*	0.5
H25	0.405761	0.246742	0.551741	0.124*	0.5
C26	0.4489 (5)	0.2677 (7)	0.573 (4)	0.253 (10)*	0.5
H26A	0.463067	0.283467	0.526681	0.303*	0.5
H26B	0.455306	0.255248	0.669565	0.303*	0.5
C24A	0.40402 (15)	0.27709 (16)	0.4069 (9)	0.0320 (9)*	0.5
H24C	0.419940	0.288767	0.478513	0.038*	0.5
H24D	0.402203	0.254619	0.448981	0.038*	0.5
C25A	0.4134 (3)	0.2777 (4)	0.2274 (16)	0.104 (3)*	0.5
H25A	0.414367	0.296477	0.157479	0.124*	0.5
C26A	0.4210 (7)	0.2454 (6)	0.171 (4)	0.253 (10)*	0.5
H26C	0.419396	0.227889	0.249686	0.303*	0.5
H26D	0.427605	0.241649	0.056278	0.303*	0.5

### Atomic displacement parameters (Å^2^)

**Table:**

	*U*^11^	*U*^22^	*U*^33^	*U*^12^	*U*^13^	*U*^23^
Cl1	0.0197 (3)	0.0175 (3)	0.0562 (5)	−0.0022 (2)	0.0040 (3)	−0.0126 (3)
Cl2	0.0226 (3)	0.0189 (3)	0.0947 (7)	−0.0046 (2)	−0.0035 (4)	−0.0100 (4)
Cl3	0.0204 (3)	0.0253 (3)	0.0245 (3)	0.0027 (2)	0.0056 (2)	0.0038 (2)
Cl4	0.0212 (3)	0.0206 (3)	0.0258 (3)	0.0057 (2)	−0.0041 (2)	0.0019 (2)
Cl5	0.0196 (3)	0.0342 (3)	0.0314 (3)	0.0135 (2)	0.0019 (2)	−0.0024 (3)
Cl6	0.0300 (3)	0.0393 (4)	0.0231 (3)	0.0109 (3)	0.0075 (3)	0.0007 (3)
O2	0.0132 (9)	0.0229 (10)	0.0844 (19)	−0.0038 (8)	0.0105 (10)	−0.0175 (11)
O3	0.0138 (8)	0.0161 (8)	0.0617 (14)	0.0014 (6)	0.0016 (9)	−0.0128 (9)
O4	0.0264 (10)	0.0162 (9)	0.098 (2)	0.0031 (8)	−0.0117 (12)	−0.0170 (11)
C1	0.0125 (11)	0.0202 (12)	0.0529 (18)	−0.0005 (9)	0.0033 (11)	−0.0103 (12)
C2	0.0184 (12)	0.0158 (11)	0.0400 (15)	−0.0010 (9)	0.0018 (11)	−0.0095 (11)
C3	0.0127 (10)	0.0182 (11)	0.0328 (14)	0.0008 (8)	0.0015 (10)	−0.0071 (10)
C4	0.0142 (10)	0.0156 (11)	0.0268 (12)	0.0018 (8)	0.0008 (9)	−0.0052 (9)
C5	0.0168 (11)	0.0151 (11)	0.0407 (15)	0.0024 (9)	−0.0002 (11)	−0.0082 (10)
C6	0.0139 (11)	0.0220 (13)	0.0579 (19)	0.0017 (9)	0.0042 (12)	−0.0120 (13)
C7	0.0226 (13)	0.0154 (12)	0.0580 (19)	0.0035 (10)	−0.0084 (13)	−0.0062 (12)
C8	0.0206 (13)	0.0177 (12)	0.066 (2)	0.0050 (10)	−0.0058 (13)	−0.0106 (13)
C9	0.0146 (11)	0.0165 (11)	0.0422 (15)	0.0014 (9)	−0.0040 (10)	−0.0049 (11)
C10	0.0155 (11)	0.0139 (10)	0.0242 (12)	0.0023 (8)	−0.0033 (9)	−0.0014 (9)
C11	0.0154 (11)	0.0173 (11)	0.0305 (13)	0.0012 (9)	−0.0032 (10)	−0.0004 (10)
C12	0.0193 (12)	0.0146 (11)	0.0447 (16)	−0.0013 (9)	−0.0071 (11)	−0.0033 (11)
C13	0.0133 (10)	0.0161 (11)	0.0215 (11)	0.0013 (8)	−0.0024 (9)	−0.0009 (9)
C14	0.0120 (10)	0.0116 (10)	0.0238 (12)	−0.0012 (8)	−0.0011 (9)	−0.0043 (9)
C15	0.0124 (10)	0.0131 (10)	0.0231 (12)	−0.0020 (8)	0.0003 (9)	−0.0018 (9)
C16	0.0147 (10)	0.0122 (10)	0.0231 (12)	0.0003 (8)	−0.0041 (9)	−0.0014 (9)
C17	0.0130 (10)	0.0173 (11)	0.0256 (12)	0.0015 (8)	−0.0007 (9)	−0.0053 (9)
C18	0.0175 (11)	0.0221 (12)	0.0199 (12)	0.0016 (9)	0.0023 (9)	−0.0052 (9)
C19	0.0184 (11)	0.0170 (11)	0.0222 (12)	0.0034 (9)	−0.0018 (9)	−0.0033 (9)

### Geometric parameters (Å, º)

**Table:**

Cl1—C2	1.732 (2)	C19—C20A	1.511 (6)
Cl2—C12	1.722 (3)	C19—C20B	1.533 (7)
Cl3—C15	1.714 (2)	O5A—C20A	1.162 (8)
Cl4—C16	1.718 (2)	O6A—C20A	1.343 (8)
Cl5—C17	1.715 (2)	O6A—C21A	1.450 (9)
Cl6—C18	1.718 (3)	C21A—H21A	0.9900
O2—H2	0.74 (4)	C21A—H21B	0.9900
O2—C1	1.326 (3)	C21A—C22A	1.427 (10)
O3—C5	1.364 (3)	C22A—H22A	0.9500
O3—C9	1.367 (3)	C22A—C23A	1.355 (11)
O4—C7	1.251 (3)	C23A—H23A	0.9500
C1—C2	1.420 (3)	C23A—H23B	0.9500
C1—C6	1.389 (4)	O5B—C20B	1.188 (8)
C2—C3	1.364 (3)	O6B—C20B	1.329 (9)
C3—H3	0.9500	O6B—C21B	1.486 (10)
C3—C4	1.412 (3)	C21B—H21C	0.9900
C4—C5	1.403 (3)	C21B—H21D	0.9900
C4—C13	1.425 (3)	C21B—C22B	1.407 (18)
C5—C6	1.381 (4)	C22B—H22B	0.9500
C6—H6	0.9500	C22B—C23B	1.411 (15)
C7—C8	1.432 (4)	C23B—H23C	0.9500
C7—C12	1.459 (4)	C23B—H23D	0.9500
C8—C9	1.359 (4)	C24—H24A	0.9900
C8—C24	1.552 (7)	C24—H24B	0.9900
C8—C24A	1.519 (6)	C24—C25	1.531 (8)
C9—C10	1.441 (3)	C25—H25	0.9500
C10—C11	1.429 (3)	C25—C26	1.385 (17)
C10—C13	1.373 (3)	C26—H26A	0.9500
C11—H11	0.9500	C26—H26B	0.9500
C11—C12	1.347 (3)	C24A—H24C	0.9900
C13—C14	1.499 (3)	C24A—H24D	0.9900
C14—C15	1.393 (3)	C24A—C25A	1.451 (12)
C14—C19	1.389 (3)	C25A—H25A	0.9500
C15—C16	1.392 (3)	C25A—C26A	1.442 (17)
C16—C17	1.387 (3)	C26A—H26C	0.9500
C17—C18	1.395 (4)	C26A—H26D	0.9500
C18—C19	1.397 (3)		
			
C1—O2—H2	110 (3)	C14—C19—C20B	120.3 (3)
C5—O3—C9	121.41 (19)	C18—C19—C20A	123.2 (3)
O2—C1—C2	117.5 (2)	C18—C19—C20B	119.6 (3)
O2—C1—C6	123.9 (2)	C20A—O6A—C21A	118.2 (6)
C6—C1—C2	118.6 (2)	O5A—C20A—C19	126.1 (6)
C1—C2—Cl1	118.09 (19)	O5A—C20A—O6A	126.5 (6)
C3—C2—Cl1	120.30 (19)	O6A—C20A—C19	107.4 (5)
C3—C2—C1	121.6 (2)	O6A—C21A—H21A	109.6
C2—C3—H3	119.9	O6A—C21A—H21B	109.6
C2—C3—C4	120.2 (2)	H21A—C21A—H21B	108.1
C4—C3—H3	119.9	C22A—C21A—O6A	110.1 (6)
C3—C4—C13	124.0 (2)	C22A—C21A—H21A	109.6
C5—C4—C3	117.5 (2)	C22A—C21A—H21B	109.6
C5—C4—C13	118.4 (2)	C21A—C22A—H22A	116.8
O3—C5—C4	121.0 (2)	C23A—C22A—C21A	126.3 (10)
O3—C5—C6	116.3 (2)	C23A—C22A—H22A	116.8
C6—C5—C4	122.6 (2)	C22A—C23A—H23A	120.0
C1—C6—H6	120.3	C22A—C23A—H23B	120.0
C5—C6—C1	119.4 (2)	H23A—C23A—H23B	120.0
C5—C6—H6	120.3	C20B—O6B—C21B	116.7 (6)
O4—C7—C8	121.0 (3)	O5B—C20B—C19	120.6 (6)
O4—C7—C12	121.8 (2)	O5B—C20B—O6B	127.4 (6)
C8—C7—C12	117.2 (2)	O6B—C20B—C19	112.0 (5)
C7—C8—C24	118.4 (3)	O6B—C21B—H21C	109.0
C7—C8—C24A	117.1 (3)	O6B—C21B—H21D	109.0
C9—C8—C7	119.4 (2)	H21C—C21B—H21D	107.8
C9—C8—C24	121.5 (3)	C22B—C21B—O6B	112.7 (9)
C9—C8—C24A	122.5 (3)	C22B—C21B—H21C	109.0
O3—C9—C10	119.4 (2)	C22B—C21B—H21D	109.0
C8—C9—O3	117.1 (2)	C21B—C22B—H22B	119.5
C8—C9—C10	123.4 (2)	C21B—C22B—C23B	121.0 (17)
C11—C10—C9	117.0 (2)	C23B—C22B—H22B	119.5
C13—C10—C9	119.4 (2)	C22B—C23B—H23C	120.0
C13—C10—C11	123.6 (2)	C22B—C23B—H23D	120.0
C10—C11—H11	119.8	H23C—C23B—H23D	120.0
C12—C11—C10	120.5 (2)	C8—C24—H24A	110.8
C12—C11—H11	119.8	C8—C24—H24B	110.8
C7—C12—Cl2	117.33 (19)	H24A—C24—H24B	108.8
C11—C12—Cl2	120.1 (2)	C25—C24—C8	105.0 (7)
C11—C12—C7	122.5 (2)	C25—C24—H24A	110.8
C4—C13—C14	118.5 (2)	C25—C24—H24B	110.8
C10—C13—C4	120.3 (2)	C24—C25—H25	115.9
C10—C13—C14	121.2 (2)	C26—C25—C24	128.1 (18)
C15—C14—C13	119.0 (2)	C26—C25—H25	115.9
C19—C14—C13	121.6 (2)	C25—C26—H26A	120.0
C19—C14—C15	119.4 (2)	C25—C26—H26B	120.0
C14—C15—Cl3	119.32 (17)	H26A—C26—H26B	120.0
C16—C15—Cl3	120.06 (19)	C8—C24A—H24C	110.8
C16—C15—C14	120.6 (2)	C8—C24A—H24D	110.8
C15—C16—Cl4	120.03 (19)	H24C—C24A—H24D	108.9
C17—C16—Cl4	120.14 (18)	C25A—C24A—C8	104.6 (7)
C17—C16—C15	119.8 (2)	C25A—C24A—H24C	110.8
C16—C17—Cl5	120.01 (19)	C25A—C24A—H24D	110.8
C16—C17—C18	119.8 (2)	C24A—C25A—H25A	125.3
C18—C17—Cl5	120.13 (19)	C26A—C25A—C24A	109.5 (17)
C17—C18—Cl6	119.82 (18)	C26A—C25A—H25A	125.3
C17—C18—C19	120.1 (2)	C25A—C26A—H26C	120.0
C19—C18—Cl6	120.0 (2)	C25A—C26A—H26D	120.0
C14—C19—C18	120.0 (2)	H26C—C26A—H26D	120.0
C14—C19—C20A	115.8 (3)		
			
Cl1—C2—C3—C4	178.0 (2)	C9—C10—C13—C4	−0.3 (4)
Cl3—C15—C16—Cl4	−3.2 (3)	C9—C10—C13—C14	176.7 (2)
Cl3—C15—C16—C17	176.60 (17)	C10—C11—C12—Cl2	−178.5 (2)
Cl4—C16—C17—Cl5	−2.3 (3)	C10—C11—C12—C7	0.6 (4)
Cl4—C16—C17—C18	178.46 (18)	C10—C13—C14—C15	−104.3 (3)
Cl5—C17—C18—Cl6	0.0 (3)	C10—C13—C14—C19	75.6 (3)
Cl5—C17—C18—C19	−176.96 (19)	C11—C10—C13—C4	−178.3 (2)
Cl6—C18—C19—C14	−176.83 (18)	C11—C10—C13—C14	−1.4 (4)
Cl6—C18—C19—C20A	−8.2 (5)	C12—C7—C8—C9	−0.2 (5)
Cl6—C18—C19—C20B	5.8 (5)	C12—C7—C8—C24	−170.4 (4)
O2—C1—C2—Cl1	0.1 (4)	C12—C7—C8—C24A	168.5 (4)
O2—C1—C2—C3	178.9 (3)	C13—C4—C5—O3	−2.5 (4)
O2—C1—C6—C5	−178.4 (3)	C13—C4—C5—C6	176.1 (3)
O3—C5—C6—C1	178.6 (3)	C13—C10—C11—C12	177.5 (3)
O3—C9—C10—C11	178.5 (2)	C13—C14—C15—Cl3	5.6 (3)
O3—C9—C10—C13	0.3 (4)	C13—C14—C15—C16	−175.8 (2)
O4—C7—C8—C9	179.0 (3)	C13—C14—C19—C18	176.7 (2)
O4—C7—C8—C24	8.8 (6)	C13—C14—C19—C20A	7.3 (4)
O4—C7—C8—C24A	−12.3 (6)	C13—C14—C19—C20B	−6.0 (5)
O4—C7—C12—Cl2	−0.2 (5)	C14—C15—C16—Cl4	178.20 (17)
O4—C7—C12—C11	−179.4 (3)	C14—C15—C16—C17	−2.0 (3)
C1—C2—C3—C4	−0.7 (4)	C14—C19—C20A—O5A	58.7 (7)
C2—C1—C6—C5	1.1 (5)	C14—C19—C20A—O6A	−120.2 (4)
C2—C3—C4—C5	1.7 (4)	C14—C19—C20B—O5B	61.9 (7)
C2—C3—C4—C13	−175.6 (3)	C14—C19—C20B—O6B	−116.7 (5)
C3—C4—C5—O3	−179.9 (3)	C15—C14—C19—C18	−3.4 (3)
C3—C4—C5—C6	−1.2 (4)	C15—C14—C19—C20A	−172.9 (4)
C3—C4—C13—C10	178.5 (3)	C15—C14—C19—C20B	173.9 (4)
C3—C4—C13—C14	1.5 (4)	C15—C16—C17—Cl5	177.90 (18)
C4—C5—C6—C1	−0.1 (5)	C15—C16—C17—C18	−1.4 (3)
C4—C13—C14—C15	72.7 (3)	C16—C17—C18—Cl6	179.24 (18)
C4—C13—C14—C19	−107.4 (3)	C16—C17—C18—C19	2.3 (4)
C5—O3—C9—C8	176.9 (3)	C17—C18—C19—C14	0.1 (4)
C5—O3—C9—C10	−1.5 (4)	C17—C18—C19—C20A	168.8 (4)
C5—C4—C13—C10	1.3 (4)	C17—C18—C19—C20B	−177.2 (4)
C5—C4—C13—C14	−175.7 (2)	C18—C19—C20A—O5A	−110.4 (6)
C6—C1—C2—Cl1	−179.4 (3)	C18—C19—C20A—O6A	70.7 (5)
C6—C1—C2—C3	−0.7 (5)	C18—C19—C20B—O5B	−120.8 (6)
C7—C8—C9—O3	−178.1 (3)	C18—C19—C20B—O6B	60.6 (6)
C7—C8—C9—C10	0.1 (5)	C19—C14—C15—Cl3	−174.23 (18)
C7—C8—C24—C25	−88.3 (7)	C19—C14—C15—C16	4.4 (3)
C7—C8—C24A—C25A	94.0 (8)	O6A—C21A—C22A—C23A	−118.3 (10)
C8—C7—C12—Cl2	179.0 (3)	C20A—O6A—C21A—C22A	−88.9 (8)
C8—C7—C12—C11	−0.2 (5)	C21A—O6A—C20A—C19	−178.2 (5)
C8—C9—C10—C11	0.3 (4)	C21A—O6A—C20A—O5A	2.8 (10)
C8—C9—C10—C13	−177.9 (3)	O6B—C21B—C22B—C23B	107.2 (16)
C8—C24—C25—C26	−125 (2)	C20B—O6B—C21B—C22B	−97.9 (11)
C8—C24A—C25A—C26A	−126.4 (15)	C21B—O6B—C20B—C19	179.9 (5)
C9—O3—C5—C4	2.6 (4)	C21B—O6B—C20B—O5B	1.4 (10)
C9—O3—C5—C6	−176.1 (3)	C24—C8—C9—O3	−8.2 (5)
C9—C8—C24—C25	101.7 (7)	C24—C8—C9—C10	170.1 (4)
C9—C8—C24A—C25A	−97.7 (8)	C24A—C8—C9—O3	13.8 (6)
C9—C10—C11—C12	−0.6 (4)	C24A—C8—C9—C10	−167.9 (4)

### Hydrogen-bond geometry (Å, º)

**Table:**

*D*—H···*A*	*D*—H	H···*A*	*D*···*A*	*D*—H···*A*
O2—H2···O4^i^	0.74 (4)	1.86 (4)	2.595 (3)	172 (4)

Symmetry code: (i) *y*+1/4, −*x*+3/4, −*z*+3/4.
